# Amphibians and Reptiles of the state of Nuevo León, Mexico

**DOI:** 10.3897/zookeys.594.8289

**Published:** 2016-05-30

**Authors:** Julio A. Lemos-Espinal, Geoffrey R. Smith, Alexander Cruz

**Affiliations:** 1 Laboratorio de Ecología-UBIPRO, FES Iztacala UNAM. Avenida los Barrios 1, Los Reyes Iztacala, Tlalnepantla, edo. de México, Mexico - 54090; 2 Department of Biology, Denison University, Granville, OH, USA 43023; 3 Department of Ecology and Evolutionary Biology (EBIO), University of Colorado - Boulder, Campus Box 334 UCB, Boulder, COUSA 80309-0334

**Keywords:** Checklist, Conservation status, Herpetofauna, IUCN Red List

## Abstract

We compiled a check list of the herpetofauna of Nuevo León. We documented 132 species (23 amphibians, 109 reptiles), representing 30 families (11 amphibians, 19 reptiles) and 73 genera (17 amphibians, 56 reptiles). Only two species are endemic to Nuevo León. Nuevo León contains a relatively high richness of lizards in the genus *Sceloporus*. Overlap in the herpetofauna of Nuevo León and states it borders is fairly extensive. Of 130 native species, 102 are considered species of Least Concern in the IUCN red list, four are listed as Vulnerable, five are listed as Near Threatened, and four are listed as Endangered. According to SEMARNAT, 78 species are not of conservation concern, 25 are subject to Special Protection, 27 are Threatened, and none are listed as in Danger of Extinction. Given current threats to the herpetofauna, additional efforts to understand the ecology and status of populations in Nuevo León are needed.

## Introduction

The flora and fauna of Nuevo León is very species rich. Broadly speaking, it consists mainly of a group of species characteristic of the great deserts of North America, as well as species from the temperate forests of the Sierra Madre Oriental and subtropical species that extend their distribution northward, in some cases even from Central or South America, through the lowlands of the Atlantic slope. Despite these characteristics, there have been few studies on the diversity and distribution of the species of amphibians and reptiles in the state and those that have been conducted have focused almost entirely on the forests of the Sierra Madre Oriental and satellite mountains to the north and northeast of the city of Monterrey (Valdez-Tamez et al. 2003, Lazcano et al. 2004, 2007, 2009a, Contreras-Lozano et al. 2007, 2010, 2011a, 2012, Lemos-Espinal and Cruz 2015). The vast plains to the east of the Sierra Madre Oriental as well as the portion of the Mexican Plateau in the southwestern corner of the state remain relatively unstudied. Currently there is no systematic program to study the state’s herpetofauna across different regions. Consequently, one of the main threats to the conservation of the diversity of amphibians and reptiles of the state is a lack of knowledge. Coupling this lack of knowledge of the herpetofauna of the state with problems associated with the high demand for water, energy, and food to meet the increasing needs of one of the largest and fastest growing cities in Mexico (Monterrey) does not suggest an encouraging outlook for the future of the herpetofauna of Nuevo León. To help increase the awareness of the herpetofaunal richness of Nuevo Léon, we gathered information on the presence of amphibian and reptile species. In addition, given the potential for increased impacts of humans on the environment, we also gathered information on the conservation status of these species. Our goal is to provide a readily accessible compilation of the herpetofaunal species and their conservation status, and to expand upon previous statewide checklists (e.g., Lemos-Espinal and Cruz 2015).

Thomas H. Webb made the earliest herpetological collections in Nuevo León in 1852, as part of the Boundary Commission Survey (see Kellogg 1932). Since then herpetological surveys of Nuevo León have been conducted with varying coverage and intensity. The work of Edward Taylor and Hobart Smith contributed greatly to increasing the knowledge of the herpetofauna of the state (e.g., Smith 1934, 1942, 1944, 1951, Taylor 1939, 1940, 1941, Taylor and Smith 1945, Smith and Hall 1974). More recently, several papers, distributional records, and natural history notes have been published documenting range extensions (e.g., Salmon et al. 2001, 2004, Price et al. 2010, Price and Lazcano-Villareal 2010, Banda-Leal et al. 2002, 2014a), behavior (Contreras-Lozano et al. 2011b), body size (Banda et al. 2005, Lazcano and Bryson 2010), parasites (García-de la Peña et al. 2004, 2005, León-Regagnon et al. 2005), morphological anomalies (Chávez-Cisneros and Lazcano 2012), diet (Castañeda et al. 2005, Lazcano et al. 2006a, 2011a, Banda-Leal et al. 2014b), sexual dimorphism (García-Bastida et al. 2013), captive husbandry (Lazcano et al. 2011b), and mortality (Lazcano et al. 2006b, 2008, 2009b, Castañeda et al. 2006, Chávez Cisneros et al. 2010).

## Materials and methods

### Study site

**Figure 1.**
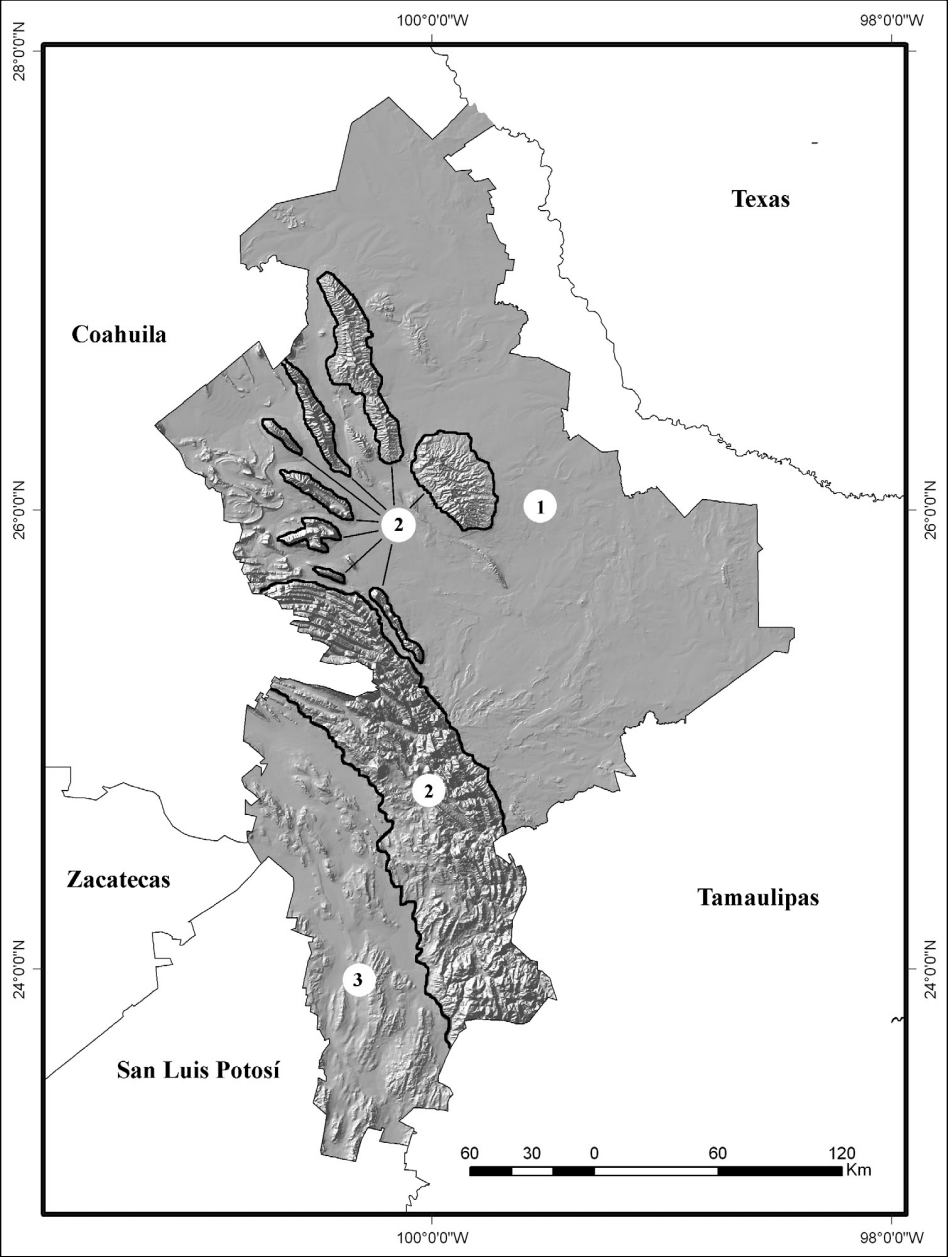
Topographical map of the state of Nuevo León, Mexico: **1** Flat Region **2** Sierra Madre Oriental, and **3** Mexican Plateau (INEGI 2001).

The State of Nuevo León is found in northeastern Mexico (98°26' to 101°14'W, 23°11' to 27°49'N). It shares its borders with the U.S. state of Texas and the Mexican states of Coahuila, Tamaulipas, San Luis Potosí, and Zacatecas (Fig. 1). The area of the state is 64,220 km^2^, with an elevational range of 50 to > 3,710 m above sea level (INEGI 2010). The capital, Monterrey, forms a large metropolitan area that contains approximately 88% of the population of Nuevo León, with more than four million inhabitants (INEGI 2010). Nuevo León has an extensive road network that runs throughout most of the state (INEGI 2010).

Three topographical regions can be identified in Nuevo León (Fig. 1; see also Lemos-Espinal and Cruz 2015 for more details). The first region (area = 23,138 km^2^; 36% of the state’s surface) is a relatively flat area with a series of small, low, scattered hills (50 to 250 m above sea level) that occurs in the central, eastern, northern, and northwestern parts of the state (Alanís-Flores et al. 1996). This region includes areas that are part of the Great Plains of North America.

The second region is the Sierra Madre Oriental, located mainly in the western portion of Nuevo León (Alanís-Flores et al. 1996). The Sierra Madre Oriental divides the low-elevation plains of the central, eastern, and northwestern parts of the state from the Mexican Plateau in the southwestern corner of the state. The Sierra Madre Oriental consists of mountain ranges that average 2000 m above sea level; with their elevation generally decreasing from south to north. The Sierra Madre Oriental is the wettest region. Near Monterrey the Sierra Madre Oriental is interrupted by valleys that create an archipelago of mountain islands (elevation not exceeding 1550 m above sea level) in the midst of arid and semiarid valleys.

The Mexican Plateau is the third region in Nuevo León, and is found in the southwestern corner of the state (Alanís-Flores et al. 1996). The altitude varies between 1500 and 2000 m, and in general is a flat and arid-semiarid area (Alanís-Flores et al. 1996, Velazco-Macías et al. 2008, Velazco-Macías 2009, INEGI 2010, Lemos-Espinal and Cruz 2015).

For most of Nuevo León, the climate is very hot, with precipitation generally not exceeding 500 mm annually (Alanís-Flores et al. 1996). Rains occur in the summer or infrequently throughout the year. The Mexican Plateau in the southwestern part of the state is also hot and very dry with annual rainfall < 200 mm. A temperate sub-humid climate is found in the Sierra Madre Oriental (Alanís-Flores et al. 1996). Here the temperature is milder (18°C–20°C) and average annual precipitation ranges from 600 to 900 mm. At high elevations (> 3000 m), alpine and subalpine climates are found. The Coastal Plain of the Gulf in the central part of the state includes tropical, sub-hot, and subhumid climates,receiving an intermediate amount of precipitation (Alanís-Flores et al. 1996).

Six vegetation types are found in Nuevo León: Chihuahuan Desert Scrub; Tamaulipan Thorn Scrub; Submontane Scrub; Montane Forest; Grassland; and Riparian, Subaquatic, and Aquatic Vegetation, as well as 11 plant communities corresponding to three floristic provinces: the Mexican Plateau, the Coastal Plain of the Northeast, and the Sierra Madre Oriental (Rzedowski 1978).

### Data collection

We obtained the list of amphibians and reptiles of the state of Nuevo León from the following sources: (1) specimens in the collections of the Laboratorio de Ecología-UBIPRO (LEUBIPRO); (2) databases from the Comisión Nacional para el Conocimiento y Uso de la Biodiversidad (National Commission for the Understanding and Use of Biodiversity; CONABIO), that were the results of various scientific projects undertaken by this institution in Nuevo Léon and also includes records from the following 28 collections: Colección de Vertebrados, Instituto de Investigaciones de Zonas Desérticas, Universidad Autónoma de San Luis Potosí
(IIZD); Colección de Vertebrados, Universidad Autónoma de Baja California
(CMMEX); Colección Herpetológica Facultad de Ciencias Biológicas, Universidad Autónoma de Nuevo León
(UANL); Colección Herpetológica, Departamento de Zoología, Escuela Nacional de Ciencias Biológicas (ENCB); Colección Herpetológica, Museo de Zoología “Alfonso L. Herrera”, Facultad de Ciencias UNAM
(MZFC-UNAM); Colección Nacional de Anfibios y Reptiles, Instituto de Biología UNAM
(CNAR); Amphibians and Reptiles Collection, University of Arizona
(UAZ); Collection of Herpetology, Amphibians and Reptiles Section, Carnegie Museum of Natural History, Pittsburgh; Collection of Herpetology, Biology Department, Tulane University, New Orleans (TU); Collection of Herpetology, Department of Vertebrate Zoology, National Museum of Natural History, Smithsonian Institution
(USNM); Collection of Herpetology, Herpetology Department (California Academy of Sciences); Collection of Herpetology, Herpetology Department, American Museum of Natural History
(AMNH); Collection of Herpetology, Herpetology Department, California Academy of Sciences
(CAS); Collection of Herpetology, Museum of Comparative Zoology, Harvard University Cambridge (MCZ); Collection of Herpetology, Museum of Vertebrate Zoology, Division of Biological Sciences, University of California Berkeley (MVZ); Collection of Herpetology, Museum of Zoology, University of Michigan Ann Arbor (UMMZ); Collection of Herpetology, Texas Cooperative Wildlife Collection, Texas A&M University
(TCWC); Collection of Herpetology, Texas Natural History Collection, University of Texas Austin (TNHC); Collection of Herpetology, University of Colorado Museum
(UCM); Collection of Herpetology, University of Illinois Museum of Natural History
(UIMNH); Division of Amphibians and Reptiles, Field Museum of Natural History
(FMNH); Ernest A. Liner Collection of Herpetology (EALCH); Fort Worth Museum of Sciences and History (FWMSH); Herpetology Section, Natural History Museum of Los Angeles County
(LACM); Louisiana State University, Museum of Life Sciences; Merriam Museum, University of Texas Arlington (UTAMM); Museum of Natural History, Division of Herpetology, Kansas University
(MNHUK); University of Nebraska
(UNO); (3) a thorough examination of the available literature on amphibians and reptiles in the state such as: Liner (1964, 1966a,b, 1991a,b, 1992a,b, 1993, 1994a,b, 1996a,b), Liner and Chaney (1986, 1987, 1990a,b, 1995a,b), Liner and Dixon (1992, 1994), Price et al. (2010), Price and Lazcano-Villarreal (2010), Rabb (1956), among others; and (4) our personal field work, primarily focused around the extreme western part of the state on the state line between Coahuila and Nuevo León. We visited this region periodically from 2002 to 2014, taking notes on the amphibians and reptiles observed during visual encounter surveys. Taxonomy and Standard English names used here are those found in Lemos-Espinal (2015). Each species was assigned to one of three floristic provinces present in Nuevo León: the Mexican Plateau, the Coastal Plain of the Northeast, and the Sierra Madre Oriental (Rzedowski 1978).

Species were included in the check list only if we were able to confirm the record, either by direct observation or through documented museum records or vouchers in the state. Species with a questionable distribution in Nuevo León, or those that are mentioned in the literature without documented support of their presence in the state were not included in our list. In addition, we recorded the conservation status of each species based on three sources: 1) the IUCN Red List 2014; 2) Environmental Viability Scores from Wilson et al. (2013a,b); 3) listing in SEMARNAT (2010). For those neighboring states for which a recent checklist exists (Coahuila: Lemos-Espinal and Smith 2007; San Luis Potosí: Lemos-Espinal and Dixon 2013; Tamaulipas: Farr 2015; and Texas: Dixon 2015), we determined the number of overlapping species.

## Results

The herpetofauna of Nuevo León includes a total of 132 species: 23 amphibians (three salamanders, 20 anurans) and 109 reptiles (six turtles, 42 lizards, 61 snakes) (Table 1; see also Lemos-Espinal and Cruz 2015). These represent 30 families: 11 of amphibians (two of salamanders and nine of frogs), and 19 of reptiles (four of turtles, eight of lizards and seven of snakes), and 73 genera: 17 of amphibians (three of salamanders and 14 of frogs), and 56 of reptiles (five of turtles, 14 of lizards and 37 of snakes) (Table 1).

**Table 1.** Checklist of amphibians and reptiles of Nuevo León. We also provide the Habitat type (CD = Chihuahuan Desert, SM = Sierra Madre Oriental, TS = Tamaulipan Thornscrub), IUCN Status (DD = Data Deficient; LC = Least Concern, V = Vulnerable, NT = Neat Threatened; E = Endangered; CE = Critically Endangered), and Environmental Vulnerability Score (EVS; the higher the score the greater the vulnerability) from Wilson et al. (2013a,b), and conservation status in Mexico according to SEMARNAT (2010) (P = in danger of extinction, A = threatened; Pr = subject to special protection, NL – not listed). Source denotes whether the species was observed in the field by the authors (A), documented in the CONABIO data base and/or museum collections (C/M), or found in the literature (citation of source).

	Habitat Type	IUCN Status	EVS Score	SEMARNAT listing	Source
Class Amphibia					
Order Caudata					
Family Ambystomatidae					
*Ambystoma mavortium* Baird	CD	?	10	NL	Reese (1971)
Family Plethodontidae					
*Chiropterotriton priscus* Rabb	SM	?	16	Pr	Rabb (1956)
*Pseudoeurycea galeanae* Taylor	SM	NT	18	A	Taylor (1941)
Order Anura					
Family Bufonidae					
*Anaxyrus cognatus* (Say)	CD	LC	9	NL	A
*Anaxyrus debilis* (Girard)	CD	LC	7	Pr	A
*Anaxyrus punctatus* (Baird & Girard)	CD	LC	5	NL	A
*Anaxyrus speciosus* (Girard)	CD	LC	12	NL	A
*Incilius nebulifer* (Girard)	TS	LC	6	NL	A
*Rhinella marina* (Linnaeus)	TS, SM	LC	3	NL	C/M
Family Craugastoridae					
*Craugastor augusti* (Dugès)	SM	LC	8	NL	C/M
Family Eleutherodactylidae					
*Eleutherodactylus cystignathoides* (Cope)	SM	LC	12	NL	C/M
*Eleutherodactylus guttilatus* (Cope)	SM	LC	11	NL	C/M
*Eleutherodactylus longipes* (Baird)	SM	V	15	NL	C/M
Family Hylidae					
*Ecnomiohyla miotympanum* (Cope)	SM	NT	9	NL	C/M
*Smilisca baudinii* (Duméril & Bibron)	SM	LC	3	NL	A
Family Leptodactylidae					
*Leptodactylus fragilis* (Brocchi)	GL, Rip (CD)	LC	5	NL	C/M
Family Microhylidae					
*Gastrophryne olivacea* (Hallowell)	CD	LC	9	Pr	A
*Hypopachus variolosus* (Cope)	SM	LC	4	NL	C/M
Family Ranidae					
*Lithobates berlandieri* (Baird)	CD	LC	7	Pr	C/M
Family Rhinophrynidae					
*Rhinophrynus dorsalis* Duméril & Bibron	TS (Tamaulipan)	LC	8	Pr	C/M
Family Scaphiopodidae					
*Scaphiopus couchii* Baird	CD	LC	3	NL	A
*Spea bombifrons* (Cope)	CD	LC	10	NL	C/M
*Spea multiplicata* (Cope)	CD	LC	6	NL	A
Class Reptilia					
Order Testudines					
Family Emidydae					
*Pseudemys gorzugi* Ward	CD	NT	16	A	C/M
*Trachemys scripta* (Thusberg)	CD	LC	16	Pr	C/M
Family Kinosternidae					
*Kinosternon flavescens* (Agassiz)	CD	LC	12	NL	C/M
*Kinosternon integrum* Le Conte	Riparian CD	LC	11	Pr	C/M
Family Testudinae					
*Gopherus berlandieri* (Agassiz)	TS	LC	18	A	C/M
Family Trionychidae					
*Apalone spinifera* (Le Sueur)	CD	LC	15	Pr	C/M
Order Squamata					
Suborder Lacertilia					
Family Anguidae					
*Barisia ciliaris* (Smith)	SM	?	15	NL	A
*Gerrhonotus infernalis* Baird	SM	LC	13	NL	C/M
*Gerrhonotus parvus* (Knight & Scudday)	SM	E	17	Pr	C/M
Family Crotaphytidae					
*Crotaphytus collaris* (Say)	CD	LC	13	A	C/M
*Crotaphytus reticulatus* Baird	TS	V	12	A	C/M
Family Eublepharidae					
*Coleonyx brevis* Stejneger	CD	LC	14	Pr	C/M
Family Gekkonidae					
*Hemidactylus turcicus* (Linneaus)	CD	N/A	N/A	N/A	C/M
Family Phrynosomatidae					
*Cophosaurus texanus* Troschel	CD	LC	14	A	A
*Holbrookia approximans* Baird	CD	?	14	NL	A
*Holbrookia lacerata* Cope	CD, TS	NT	14	A	C/M
*Phrynosoma cornutum* (Harlan)	CD	LC	11	NL	A
*Phrynosoma modestum* Girard	CD	LC	12	NL	A
*Phrynosoma orbiculare* (Linnaeus)	SM	LC	12	A	A
*Sceloporus cautus* Smith	CD	LC	15	A	C/M
*Sceloporus chaneyi* Liner & Dixon	SM	E	15	NL	C/M
*Sceloporus consobrinus* Baird & Girard	CD	?	?	NL	A
*Sceloporus couchii* Baird	CD	LC	15	NL	C/M
*Sceloporus cyanogenys* Cope	CD	?	16	NL	C/M
*Sceloporus cyanostictus* Axtell & Axtell	CD	E	13	NL	Price et al. (2010)
*Sceloporus goldmani* Smith	CD	E	15	NL	C/M
*Sceloporus grammicus* Wiegmann	SM, TS	LC	9	Pr	C/M
*Sceloporus merriami* Stejneger	CD	LC	13	NL	Price and Lazcano-Villarreal (2010)
*Sceloporus minor* Cope	SM	LC	14	NL	C/M
*Sceloporus oberon* Smith & Brown	SM	V	14	NL	A
*Sceloporus olivaceus* Smith	TS	LC	13	NL	C/M
*Sceloporus ornatus* Baird	CD	NT	16	A	C/M
*Sceloporus parvus* Smith	CD	LC	15	NL	C/M
*Sceloporus poinsettii* Baird & Girard	CD	LC	12	NL	A
*Sceloporus samcolemani* Smith & Hall	Grassland CD	LC	15	NL	C/M
*Sceloporus serrifer* Cope	SM	LC	6	NL	C/M
*Sceloporus spinosus* Wiegmann	CD	LC	12	NL	C/M
*Sceloporus torquatus* Wiegmann	SM	LC	11	NL	C/M
*Sceloporus variabilis* Wiegmann	SM	LC	5	NL	C/M
*Uta stansburiana* Baird & Girard	CD	LC	11	A	A
Family Scincidae					
*Plestiodon dicei* (Ruthven & Gaige)	SM	LC	7	NL	C/M
*Plestiodon obsoletus* (Baird & Girard)	CD	LC	11	NL	C/M
*Plestiodon tetragrammus* Baird	CD	LC	12	NL	C/M
*Scincella silvicola* (Taylor)	SM	LC	12	A	C/M
Family Teiidae					
*Aspidoscelis gularis* (Baird & Girard)	CD	LC	9	NL	C/M
*Aspidoscelis inornata* (Baird)	CD	LC	14	NL	C/M
*Aspidoscelis marmorata* (Baird & Girard)	CD	?	14	NL	C/M
Family Xantusidae					
*Lepidophyma sylvaticum* Taylor	SM	LC	11	Pr	C/M
Order Squamata					
Suborder Serpentes					
Family Colubridae					
*Arizona elegans* Kennicott	CD	LC	5	NL	C/M
*Bogertophis subocularis* (Brown)	CD	LC	14	NL	C/M
*Coluber constrictor* Linnaeus	Grassland in CD & SM	LC	10	A	C/M
*Drymarchon melanurus* (Duméril, Bibron & Duméril)	SM	LC	6	NL	C/M
*Drymobius margaritiferus* (Schlegel)	SM	?	6	NL	C/M
*Ficimia streckeri* Taylor	TS	LC	12	NL	C/M
*Gyalopion canum* Cope	CD	LC	9	NL	C/M
*Lampropeltis alterna* (Brown)	CD	LC	14	A	C/M
*Lampropeltis getula* (Blainville)	CD	LC		A	C/M
*Lampropeltis mexicana* (Garman)	SM	LC	15	A	C/M
*Lampropeltis triangulum* (Lacèpéde)	CD	?	7	A	C/M
*Leptophis mexicanus* Duméril & Bibron	SM	LC	6	A	C/M
*Masticophis flagellum* (Shaw)	CD	LC	8	A	C/M
*Masticophis schotti* Baird & Girard	CD, TS	LC	13	NL	C/M
*Masticophis taeniatus* (Hallowell)	CD	LC	10	NL	C/
*Opheodrys aestivus* (Linneaus)	SM	LC	13	NL	C/M
*Oxybelis aeneus* (Wagler)	SM	?	5	NL	C/M
*Pantherophis bairdi* (Yarrow)	CD	LC	15	NL	C/M
*Pantherophis emoryi* (Baird & Girard)	CD	LC	13	NL	C/M
*Pituophis catenifer* Blainville	CD	LC	9	NL	A
*Pituophis deppei* (Duméril)	SM	LC	14	A	C/M
*Rhinocheilus lecontei* Baird & Girard	CD	LC	8	NL	A
*Salvadora grahamiae* Baird & Girard	CD	LC	10	NL	C/M
*Senticolis triaspis* (Cope)	SM	LC	6	NL	C/M
*Sonora semiannulata* Baird & Girard	CD	LC	5	NL	C/M
*Tantilla atriceps* (Günther)	CD	LC	11	A	C/M
*Tantilla hobartsmithi* Taylor	CD	LC	11	NL	C/M
*Tantilla nigriceps* Kennicott	CD	LC	11	NL	C/M
*Tantilla rubra* Cope	SM	LC	5	Pr	C/M
*Tantilla wilcoxi* Stejneger	CD	LC	10	NL	C/M
*Trimorphodon tau* Cope	CD	LC	13	NL	C/M
Family Dipsadidae					
*Adelphicos newmanorum* Taylor	SM	LC	10	Pr	C/M
*Amastridium sapperi* (Werner)	SM	LC	10	NL	C/M
*Diadophis punctatus* (Linnaeus)	SM	LC	4	NL	C/M
*Heterodon kennerlyi* Kennicott	CD	?	11	Pr	C/M
*Hypsiglena jani* (Dugès)	CD	?	6	Pr?	C/M
*Leptodeira septentrionalis* (Kennicott)	SM	?	8	NL	C/M
*Rhadinaea montana* Smith	SM	? (E)	14	Pr (E)	Chaney and Liner (1986, 1990)
*Tropidodipsas sartorii* Cope	SM	LC	9	Pr	C/M
Family Elapidae					
*Micrurus tener* Baird & Girard	CD	LC	11	NL	C/M
Family Leptotyphlopidae					
*Rena dulcis* Baird & Girard	CD	LC	13	NL	C/M
*Rena myopica* (Garman)	SM	LC	13	NL	C/M
Family Natricidae					
*Nerodia erythrogaster* (Forster)	CD	LC	11	A	C/M
*Nerodia rhombifer* (Hallowell)	CD	LC	10	NL	C/M
*Storeria dekayi* (Holbrook)	SM	LC	7	NL	C/M
*Storeria hidalgoensis* Taylor	SM	V	13	NL	C/M
*Thamnophis cyrtopsis* (Kennicott)	CD	LC	7	A	A
*Thamnophis eques* (Reuss)	SM	LC	8	A	C/M
*Thamnophis exsul* (Baird & Girard)	SM	LC	16	NL	C/M
*Thamnophis marcianus* (Baird & Girard)	CD	LC	10	A	A
*Thamnophis proximus* (Say)	SM	LC	7	A	C/M
*Thamnophis pulchrilatus* (Cope)	SM	LC	15	NL	C/M
Family Typhlopidae					
*Indotyphlops braminus* (Daudin)		N/A	N/A	N/A	Guzmán and Muñiz-Martínez (1999)
Family Viperidae					
*Agkistrodon taylori* Burger & Robertson	SM	LC	17	A	C/M
*Crotalus atrox* Baird & Girard	CD	LC	9	Pr	A
*Crotalus lepidus* (Kennicott)	CD	LC	12	Pr	A
*Crotalus molossus* Baird & Girard	CD	LC	8	Pr	A
*Crotalus pricei* Van Denburgh	SM	LC	14	Pr	C/M
*Crotalus scutulatus* (Kennicott)	CD	LC	11	Pr	A
*Crotalus totonacus* Gloyd & Kauffeld	SM	?	17	Pr?	C/M
*Sistrurus catenatus* (Rafinesque)	CD	LC	13	Pr	C/M

**Table 2.** Summary of species present in Nuevo León by Family, Order or Suborder, and Class. Status summary indicates the number of species found in each IUCN conservation status in the Order DD, LC, V, NT, E, CE (see Table 1 for abbreviations; in some cases species have not been assigned a status by the IUCN and therefore these might not add up to the total number of species in a taxon). Mean EVS is the mean Environmental Vulnerability Score, scores ≥ 14 are considered high vulnerability (Wilson et al. 2013a,b) and conservation status in Mexico according to SEMARNAT (2010) in the Order NL, Pr, A, P (see Table 1 for abbreviations).

Class	Order/ Suborder	Family	Genera	Species	Status Summary	Mean EVS	SEMARNAT
Amphibia	Caudata		3	3	0,0,0,1,0,0	14.7	1,1,1,0
		Ambystomatidae	1	1	0,0,0,0,0,0	10	1,0,0,0
		Plethodontidae	2	2	0,0,0,1,0,0	17	0,1,1,0
	Anura		14	20	0,18,1,1,0,0	7.6	16,4,0,0
		Bufonidae	3	6	0,6,0,0,0,0	7	5,1,0,0
		Craugastoridae	1	1	0,1,0,0,0,0	8	1,0,0,0
		Eleutherodactylidae	1	3	0,2,1,0,0,0	12.7	3,0,0,0
		Hylidae	2	2	0,1,0,1,0,0	6	2,0,0,0
		Leptodactylidae	1	1	0,1,0,0,0,0	5	1,0,0,0
		Microhylidae	2	2	0,2,0,0,0,0	6.5	1,1,0,0
		Ranidae	1	1	0,1,0,0,0,0	7	0,1,0,0
		Rhynophrynidae	1	1	0,1,0,0,0,0	8	0,1,0,0
		Scaphiopodidae	2	3	0,3,0,0,0,0	6.3	3,0,0,0
	Subtotal		17	23	0,18,1,2,0,0	8.5	17,5,1,0
Reptilia							
	Testudines		5	6	0,5,0,1,0,0	14.7	1,3,2,0
		Emydidae	2	2	0,1,0,1,0,0	16	0,1,1,0
		Kinosternidae	1	2	0,2,0,0,0,0	11.5	1,1,0,0
		Testudinae	1	1	0,1,0,0,0,0	18	0,0,1,0
		Trionychidae	1	1	0,1,0,0,0,0	15	0,1,0,0
	Squamata		51	103	0,79,3,2,4,0	11.3	60,17,24,0
	Lacertilia		14	42	0,28,2,2,4,0	12.7	28,4,9,0
		Anguidae	2	3	0,1,0,0,1,0	15	2,1,0,0
		Crotaphytidae	1	2	0,1,1,0,0,0	12.5	0,0,2,0
		Eublepharidae	1	1	0,1,0,0,0,0	14	0,1,0,0
		Gekkonidae	1	1	--	--	--
		Phrynosomatidae	5	27	0,18,1,2,3,0	12.8	20,1,6,0
		Scincidae	2	4	0,4,0,0,0,0	10.5	3,0,1,0
		Teiidae	1	3	0,2,0,0,0,0	12.3	3,0,0,0
		Xantusidae	1	1	0,1,0,0,0,0	11	0,1,0,0
	Serpentes		37	61	0,51,1,0,0,0	10.3	32,13,15,0
		Colubridae	20	31	0,28,0,0,0,0	9.5	21,1,9,0
		Dipsadidae	8	8	0,4,0,0,0,0	9	3,5,0,0
		Elapidae	1	1	0,1,0,0,0,0	11	1,0,0,0
		Leptotyphlopidae	1	2	0,2,0,0,0,0	13	2,0,0,0
		Natricidae	3	10	0,9,1,0,0,0	10.4	5,0,5,0
		Typhlopidae	1	1	--	--	--
		Viperidae	3	8	0,7,0,0,0,0	12.6	0,7,1,0
	Subtotal		56	109	0,84,3,3,4,0	11.5	61,20,26,0
TOTAL			73	132	0,102,4,5,4,0		78,25,27,0

Of the 132 species we documented, two are not native to Nuevo León: the Mediterranean House Gecko (*Hemidactylus turcicus*; see Rödder and Lötters 2009), and the Brahminy Blindsnake (*Indotyphlops braminus*; see Servoss et al. 2013).Thirty-four of these 132 species are endemic to Mexico, only two, the Pygmy Alligator Lizard (*Gerrhonotus parvus*) and the Nuevo León Graceful Brown Snake (*Rhadinaea montana*), are endemic to Nuevo León, where they are found in the montane forest.

Seventeen of the 34 endemics to Mexico are limited to the highlands of the Sierra Madre Oriental (*Chiropterotriton priscus*, *Pseudoeurycea galeanae*, *Eleutherodactylus longipes*, *Ecnomiohyla miotympanum*, *Sceloporus chaneyi*, *Sceloporus minor*, *Sceloporus oberon*, *Sceloporus parvus*, *Plestiodon dice*, *Scincella silvicola*, *Lepidophyma sylvaticum*, *Pituophis deppei*, *Storeria hidalgoensis*, *Rena myopica*, *Thamnophis exsul*, *Agkistrodon taylori*, and *Crotalus totonacus*). Five of these species have a narrow distribution in southeastern – eastern Coahuila and adjacent Nuevo León (*Chiropterotriton priscus*, *Pseudoeurycea galeanae*, *Sceloporus oberon*, *Thamnophis exsul*), and even Tamaulipas (*Plestiodon dicei*). One more is limited to Nuevo León and adjacent Tamaulipas (*Sceloporus chaneyi*), and another 10 range from Nuevo León and Tamaulipas southward to southern Veracruz and northern Oaxaca, mainly on the Atlantic slopes of the Sierra Madre Oriental (*Eleutherodactylus longipes*, *Ecnomiohyla miotympanum*, *Sceloporus minor*, *Sceloporus parvus*, *Scincella silvicola*, *Lepidophyma sylvaticum*, *Storeria hidalgoensis*, *Rena myopica*, *Agkistrodon taylori*, and *Crotalus totonacus*). One other, *Pituophis deppei*, occurs in the Sierra Madre Oriental, the Mexican Plateau, the Transvolcanic Belt, and the Sierra Madre Occidental.

Two of the remaining 15 Mexican endemic species are limited to Coahuila and extreme western Nuevo León (*Sceloporus cyanosticus* and *Sceloporus ornatus*). Two more species are limited to scattered regions of northern Mexico: *Sceloporus couchii* to the northern Sierras of Coahuila and central western Nuevo León; and *Sceloporus goldmani* to a small area in southeastern Coahuila, adjacent Nuevo León and northeastern San Luis Potosí. Three more species endemic to Mexico are limited to the Mexican Plateau (*Sceloporus cautus*, *Sceloporus samcolemani*, and *Lampropeltis mexicana*). Four additional species (*Kinosternon integrum*, *Sceloporus spinosus*, *Sceloporus torquatus*, and *Trimorphodon tau*) are widely distributed from central Mexico through the Mexican Plateau, and in some cases (*Trimorphodon tau*) on both coasts. One species has disjunct populations in the highlands of Mexico (*Thamnophis pulchrilatus*), and another is limited to the Chihuahuan Desert of Mexico (*Holbrookia approximans*). The other two species endemic to Mexico (*Barisia imbricata* and *Phrynosoma orbiculare*), occur on the Sierra Madre Occidental and the Sierra Madre Oriental; one of them, *Phrynosoma orbiculare*, ranges even into the mountains of the Transvolcanic Belt of the central part of the country.

Of the 130 native species of amphibians and reptiles in Nuevo León, 102 are considered species of Least Concern in the IUCN red list (18 amphibians, 84 reptiles), four species (1 amphibian, 3 reptiles) are listed as Vulnerable, five species (2 amphibians, 3 reptiles) are listed as Near Threatened, and four species (0 amphibians, 4 reptiles) are listed as Endangered (IUCN 2015). Also, according to SEMARNAT listing (SEMARNAT 2010), 78 species are not listed (i.e., not of conservation concern; 17 amphibians, 61 reptiles), 25 species are Subject to Special Protection (5 amphibians, 20 reptiles), 27 species are Threatened (1 amphibian, 26 reptiles), and no species are listed as in Danger of Extinction. Several taxa are also at risk according to the EVS values. In particular, plethodontid salamanders, turtles in the families Emydidae and Testudinae, and anguid lizards have high EVS values, suggesting they may be of particular concern.

## Discussion

Nuevo León does not have a particularly large number of amphibian and reptile species, at least compared to some other states in Mexico (Ranges: amphibians 5 – 140, mean ± 1 S.E. = 36.0 ± 5.1; reptiles 31 – 263, mean ± 1 S.E. = 105.4 ± 8.9; Total 47 – 403, mean ± 1 S.E. = 141.5 ± 13.7; see Flores-Villela and García-Vázquez 2014, Parra-Olea et al. 2014). There are relatively few species endemic to Nuevo León (only two). However, Nuevo Léon has a relatively high richness of lizards in the genus *Sceloporus* (N = 20, 15.3% of all species of amphibians and reptiles found in Nuevo León).

The amount of overlap in the herpetofauna of Nuevo León and the states it borders is fairly extensive. The greatest overlap is with Tamaulipas, with the two states sharing 82.3% of Nuevo León’s herpetofauna, and especially its amphibians (95.7%) and to a lesser extent, its reptiles (79.4%). Nuevo León shares 79.2% of its herpetofauna with Coahuila (78.2% amphibians, 79.4% reptiles). Of Nuevo León’s herpetofauna, 71.5% is shared with San Luis Potosí (78.2% amphibians, 70.1% reptiles). The state with the least overlap is Texas (overall 65.4%, amphibians 82.6%, reptiles 61.7%), which might be expected since the extent of the border is relatively small and they are separated by the Río Grande. In an analysis of the herpetofauna of the Mexican and United States Border States, including Nuevo León, Smith and Lemos-Espinal (2015) found significant similarities or clustering among Texas, Tamaulipas, Nuevo León, and Coahuila for the entire herpetofauna, with subsets of the herpetofauna (e.g., amphibians, reptiles, anurans, and lizards) showing slightly different clustering patterns; although Nuevo León frequently clustered with Tamaulipas and Coahuila. The particular similarity among Nuevo León, Tamaulipas, and Coahuila likely reflects similarities in the biotic provinces found in those states (Smith and Lemos-Espinal 2015).

The conservation status of the herpetofauna of Nuevo León has relatively fewer species listed in the IUCN red list and SEMARNAT compared to other states, at least as far as listings based on IUCN red list and SEMARNAT, as well as the EVS values provided in Wilson et al. (2013a, b). This is not to say that the herpetofauna of Nuevo León should be considered safe. Given the expanding population of Monterrey, and the potential consequences of such expansion on natural resources in the state, we must be vigilant to the threats to the herpetofauna of Nuevo León. In addition, the relative paucity of ecological studies on the amphibians and reptiles in Nuevo León mean much of our understanding of these species’ status in Nuevo León is based upon information obtained in other states. We hope our summary of the species of amphibians and reptiles from Nuevo León, and their putative conservation status, will prompt further research on the ecology and status of these species in Nuevo León. Such baseline information is critical to evaluating and monitoring any changes in populations in Nuevo León that can arise due to urbanization around Monterrey and potential climate change and other threats in this area (see Biggs et al. 2010, Lavín-Murcio and Lazcano 2010, Seager and Vecchi 2010).

## References

[B1] Alanís-Flores GJ Cano y Cano G Rovalo-Merino M (1996) Vegetación y flora de Nuevo León, una guía botánico-ecológica. Impresora Monterrey, México, 265 pp.

[B2] Banda J Bryson RW Jr Lazcano D (2005) *Gerrhonotus parvus*. Maximum size. Herpetological Review 36: 449.

[B3] Banda-Leal J Bryson RW Jr Lazcano-Villarreal D (2002) New record of *Elgaria parva* (Lacertilia: Anguidae) from Nuevo León, Mexico. Southwestern Naturalist 47: 614–615. doi: 10.2307/3672668

[B4] Banda-Leal J Lazcano D Nevárez-de los Reyes M Barriga-Vallejo C (2014a) *Gerrhonotus parvus* Kinght & Scudday, 1985 (Squamata: Anguidae): New range extension and clutch size in the state of Nuevo León, Mexico. Check List 10(4): 950–953. http://biotaxa.org/cl/article/view/10.4.950/9750

[B5] Banda-Leal J Lazcano D Nevárez-de los Reyes M Barriga-Vallejo C Aguilera C Luna-Peña SA (2014b) *Ambystoma velasci*. Predation. Herpetological Review 45: 675–676.

[B6] Biggs TW Atkinson E Powell R Ojeda-Revah L (2010) Land cover following rapid urbanization on the US-Mexico border: Implications for conceptual models of urban watershed processes. Landscape and Urban Planning 96: 78–87 doi: 10.1016/j.landurbplan.2010.02.005

[B7] Castañeda G García de la Peña C Lazcano D Banda-Leal J (2005) *Cophosaurus texanus*. Saurophagy. Herpetological Review 36: 174.

[B8] Castañeda G Lazcano D García-de la Peña C Contreras-Lozano JA (2006) *Sceloporus cyanogenys*. Predation. Herpetological Review 37: 227.

[B9] Chaney AH Liner EA (1986) Geographic Distribution: *Rhadinaea montana*. Herpetological Review 17: 67

[B10] Chaney AH Liner EA (1990) Geographic Distribution: *Rhadinaea montana*. Herpetological Review 21: 23–24.

[B11] Chávez-Cisneros JA Lazcano D (2012) *Sceloporus marmoratus*. Kyphosis and scoliosis. Herpetological Review 43: 140.

[B12] Chávez-Cisneros JA Lazcano D Salinas Camarena MA (2010) *Cophosaurus texanus*. Mortality. Herpetological Review 41: 75.

[B13] Contreras-Lozano JA Lazcano D Contreras-Balderas AJ (2011a) Distribucion ecologica de la herpetofauna en gradients altitudinales superiores del Cerro el Potosí, Galeana, Nuevo León, México. Acta Zoologica Mexicana 27: 231–243. http://www1.inecol.edu.mx/azm/AZM27(2)-2011/02-%20Contreras-Lozano.pdf

[B14] Contreras-Lozano JA Lazcano D Contreras-Balderas AJ (2011b) Aggregation of *Sceloporus minor* (Sauria: Phrynosomatidae) from Cerro el Potosí, Nuevo León, Mexico. Southwestern Naturalist 56: 119–120 doi: 10.1894/PAS-10.1

[B15] Contreras-Lozano JA Lazcano D Contreras-Balderas AJ (2012) Herpetofauna of the Cerro El Potosí Natural Protected Area of Nuevo León, Mexico: Status of the ecological and altitudinal distribution. Natural Areas Journal 32: 377–385 doi: 10.3375/043.032.0405

[B16] Contreras-Lozano JA Lazcano-Villarreal D Contreras-Balderas AJ (2007) Notes on Mexican herpetofauna 10: The herpetofauna of three plant communities in the Sierra de Picachos, Nuevo León, Mexico. Bulletin of the Chicago Herpetological Society 42: 177–182. http://www.chicagoherp.org/bulletin/42(11).pdf

[B17] Contreras-Lozano JA Lazcano-Villarreal D Contreras-Balderas AJ Lavín-Murcio PA (2010) Notes on Mexican herpetofauna 14: An update to the herpetofauna of Cerro El Potosí, Galeana, Nuevo León, Mexico. Bulletin of the Chicago Herpetological Society 45: 41–46 http://www.chicagoherp.org/bulletin/45(3).pdf

[B18] Dixon JR (2015) Herpetofauna of Texas. In: Lemos-Espinal JA (Ed.) Amphibians and Reptiles of the US-Mexico Border States. Texas A&M University Press, College Station, 181–195.

[B19] Farr W (2015) Herpetofauna of Tamaulipas. In: Lemos-Espinal JA (Ed.) Amphibians and Reptiles of the US-Mexico Border States. Texas A&M University Press, College Station, 101–121.

[B20] Flores-Villela O García-Vázquez UO (2014) Biodiversidad de reptiles en México. Revista Mexicana de Biodiversidad 85 (Suppl.): S467–S475. doi: 10.755-/rmb.43236

[B21] García-Bastida M Lazcano D McBrayer LD Mercado-Hernández R (2013) Sexual dimorphism in the Alligator lizard *Gerrhonotus infernalis* (Sauria: Anguidae): Implications for sexual selection. Southwestern Naturalist 58: 202–208. doi: 10.1894/0038-4909-58.2.202

[B22] García de la Peña C Castañeda GG Lazcano D (2005) *Sceloporus olivaceus*. Ectoparasitism. Herpetological Review 36: 183.

[B23] García de la Peña C Contreras-Balderas A Castañeda GG Lazcano D (2004) Infestacion y distribucion corporal de la nigua *Entrombicula alfreddugesi* (Acari: Trombiculidae) en el lacertilio de las rocas *Sceloporus couchii* (Sauria: Phrynosomatidae). Acta Zoologica Mexicana 20: 159–165 http://www1.inecol.edu.mx/azm/documents/20_2/J-Gracia-de-la-Peña.pdf

[B24] Guzmán AF Muñiz-Martínez R (1999) Primer registro de *Ramphotyphlops braminus* (Daudin 1803) (Reptilia: Typhlopidae) para el Estado de Durango, México. Vertebrata Mexicana 5: 1–3.

[B25] INEGI (Instituto Nacional de Estadística y Geografía) (2001) Modelo Digital de Terreno. Escala 1: 250,000. INEGI, Mexico City.

[B26] INEGI (Instituto Nacional de Geografía, Estadística e Informática—Dirección General de Geografía) (2010) Anuario estadístico: Nuevo León. INEGI, Mexico City.

[B27] IUCN (2014) IUCN Red List of Threatened Species, Version 2014.1. IUCN 2014, IUCN Red List of Threatened Species.

[B28] Kellogg R (1932) Mexican tailless amphibians in the United States National Museum. Bulletin of the United States National Museum 160: 1–224. doi: 10.5479/si.03629236.160.i

[B29] Lavín-Murcio PA Lazcano D (2010) Geographic distribution and conservation of the herpetofauna of northern Mexico. In: Wilson LD Townsend JH Johnson JD (Eds) Conservation of Mesoamerican Amphibians and Reptiles. Eagle Mountain Publishing, Eagle Mountain, Utah, 275–301.

[B30] Lazcano D Bryson RW Jr (2010) *Gerrhonotus parvus*. Juvenile size. Herpetological Review 41: 79.

[B31] Lazcano D Chávez-Cisneros JA Banda-Leal J (2011a) *Arizona elegans elegans*. Diet. Herpetological Review 42: 610.

[B32] Lazcano D Salinas-Camarena MA Contreras-Lozano JA (2009b) Notes on Mexican herpetofauna 12: Are roads in Nuevo León, Mexico, taking their toll on snake populations? Bulletin of the Chicago Herpetological Society 44: 69–75. http://www.chicagoherp.org/bulletin/44(5).pdf

[B33] Lazcano D Galvan RDJ García de la Peña C Castañeda GG (2006b) *Phrynosoma cornutum*. Mortality. Herpetological Review 37: 91.

[B34] Lazcano D Kardon A Muscher RJ Contreras-Lozano JA (2011b) Notes on Mexican herpetofauna 16: Captive Husbandry-Propagation of the Exile Mexican Garter Snake, *Thamnophis exsul* Rossman, 1969. Bulletin of the Chicago Herpetological Society 46: 13–17. http://www.chicagoherp.org/bulletin/46(2).pdf

[B35] Lazcano D Kardon A Recchio I Rodríguez C (2008) *Sceloporus olivaceus*. Predation. Herpetological Review 39: 94.

[B36] Lazcano D Contreras-Lozano JA Contreras-Balderas AJ Castañeda G García de la Peña C (2006a) *Sceloporus couchi*. Saurophagy. Herpetological Review 37: 227.

[B37] Lazcano D Contreras-Lozano AJA Gallardo-Valdez J García de la Peña C Castañeda G (2009a) Notes on Mexican herpetofauna 11: Herpetological diversity in Sierra “Cerro de la Silla” (Saddleback Mountain), Nuevo León, Mexico: Preliminary list. Bulletin of the Chicago Herpetological Society 44: 21–27. http://www.chicagoherp.org/bulletin/44(2).pdf

[B38] Lazcano D Sánchez-Almazán A García de la Peña C Castañeda G Contreras-Balderas A (2007) Notes on Mexican herpetofauna 9: Herpetofauna of a fragmented *Juniperus* forest in the State Natural Protected Area of San Juan y Puentes, Aramberri, Nuevo León, Mexico: Preliminary list. Bulletin of the Chicago Herpetological Society 42: 21–26. http://www.chicagoherp.org/bulletin/42(1).pdf

[B39] Lazcano D Contreras-Balderas A González-Rojas JI Castañeda G García de la Peña C Solis-Rojas C (2004) Notes on Mexican herpetofauna 6: Herpetofauna of Sierra San Antonio Peña Nevada, Zaragoza, Nuevo León, Mexico: Preliminary List. Bulletin of the Chicago Herpetological Society 39: 181–187.

[B40] Lemos-Espinal JA (Ed.) (2015) Amphibians and Reptiles of the US-Mexico Border States. Texas A&M University Press, College Station, 614 pp.

[B41] Lemos-Espinal JA Cruz A (2015) Herpetofauna of Nuevo León. In: Lemos-Espinal JA (Ed.) Amphibians and Reptiles of the US-Mexico Border States. Texas A&M University Press, College Station, 83–100.

[B42] Lemos-Espinal JA Dixon JR (2013) Amphibians and Reptiles of San Luis Potosí. Eagle Mountain Publishing, Eagle Mountain, 300 pp.

[B43] Lemos-Espinal JA Smith HM (2007) Amphibians and Reptiles of the State of Coahuila, Mexico. Universidad Nacional Autónoma de México/Comisión Nacional para el Conocimiento y Uso de la Biodiversidad, México, 550 pp.

[B44] León-Regagnon V Martínez-Salazar EA Lazcano-Villareal D Rosas-Valdez R (2005) Helminth parasites of four species of anurans from Nuevo León, Mexico. Southwestern Naturalist 50: 251–258. doi: 10.1894/0038-4909(2005)050[0251:HPOFSO]2.0.C0;2

[B45] Liner EA (1964) Notes on four small herpetological collections from Mexico. I, Introduction: Turtles and snakes. Southwestern Naturalist 8: 221–227. doi: 10.2307/3669634

[B46] Liner EA (1966a) Notes on four small herpetological collections from Mexico. II, Amphibians. Southwestern Naturalist 11: 296–312. doi: 10.2307/3669651

[B47] Liner EA (1966b) Notes on four small herpetological collections from Mexico. III, Lizards. Southwestern Naturalist 11: 406–414. doi: 10.2307/3669481

[B48] Liner EA (1991a) Mexico bound. Gulf Coast Herpetological Society Newsletter (July): 12–15.

[B49] Liner EA (1991b) Mexico bound II. Gulf Coast Herpetological Society Newsletter 1: 4–8.

[B50] Liner EA (1992a) Mexico bound III. Gulf Coast Herpetological Society Newsletter 2: 12–21.

[B51] Liner EA (1992b) Mexico bound IV. Gulf Coast Herpetological Society Newsletter 3: 5–7.

[B52] Liner EA (1993) Mexico bound V. Gulf Coast Herpetological Society Newsletter (July): 3–5.

[B53] Liner EA (1994a) Mexico bound VI. Part two. Gulf Coast Herpetological Society Newsletter 5: 2–3.

[B54] Liner EA (1994b) *Sceloporus chaneyi*. Catalogue of American Amphibians and Reptiles 588: 1.

[B55] Liner EA (1996a) Herpetological type material from Nuevo León, Mexico. Bulletin of the Chicago Herpetological Society 31: 168–171.

[B56] Liner EA (1996b) *Rhadinaea montana* Smithson. Catalogue of American Amphibians and Reptiles 640: 1–2.

[B57] Liner EA Chaney AH (1986) *Crotalus lepidus lepidus*. Reproduction. Herpetological Review 17: 89.

[B58] Liner EA Chaney AH (1987) Natural history notes: *Rhadinaea montana*. Habitat. Herpetological Review 18: 37.

[B59] Liner EA Chaney AH (1990a) Geographic distribution: *Sceloporus torquatus mikeprestoni*. Herpetological Review 21: 22–23.

[B60] Liner EA Chaney AH (1990b) *Tantilla rubra rubra*. Arboreality. Herpetological Review 21: 20.

[B61] Liner EA Chaney AH (1995a) Geographic distribution: *Cnemidophorus inornatus inornatus*. Herpetological Review 26: 154–155.

[B62] Liner EA Chaney AH (1995b) Geographic distribution: *Cnemidophorus inornatus paulus*. Herpetological Review 26: 155.

[B63] Liner EA Dixon JR (1992) A new species of the *Sceloporus scalaris* group from Cerro Peña Nevada, Nuevo León, México. Texas Journal of Science 44: 421–427.

[B64] Liner EA Dixon JR (1994) *Sceloporus chaneyi*. Catalogue of American Amphibians and Reptiles 588: 1.

[B65] Parra-Olea G Flores-Villela O Mendoza-Almeralla C (2014) Biodiversidad de anfibios en México. Revista Mexicana de Biodiversidad 85 (Suppl.): S460–S466. doi: 10.7550/rmb.32027

[B66] Price M Lazcano-Villarreal D (2010) Geographic Distribution: *Sceloporus merriami australis*. Herpetological Review 41: 109.

[B67] Price M Harrison CR Lazcano-Villarreal D (2010) Geographic Distribution: *Sceloporus cyanostictus*. Herpetological Review 41: 108.

[B68] Rabb GB (1956) A new plethodontid salamander from Nuevo León, Mexico. Fieldiana, Zoology 39: 11–20. doi: 10.5962/bhl.title.2874

[B69] Reese RW (1971) Notes on a small herpetological collection from northeastern Mexico. Journal of Herpetology 5: 67–69. doi: 10.2307/1562852

[B70] Rödder D Lötters S (2009) Niche shifts versus niche conservatism? Climatic characteristics of the native and invasive ranges of the Mediterranean house gecko (*Hemidactylus turcicus*). Global Ecology and Biogeography 18: 674–687. doi: 10.1111/j.1466-8238.2009.00477.x

[B71] Rzedowski J (1978) Vegetación de México. Editorial Limusa, México, 432 pp.

[B72] Salmon GT Bryson RW Jr Lazcano D (2001) Geographic Distribution: *Lampropeltis mexicana*. Herpetological Review 32: 1–123.

[B73] Salmon GT Liner EA Forks JE Lazcano D (2004) Geographic Distribution: *Lampropeltis alterna*. Herpetological Review 35: 292.

[B74] Seager R Vecchi GA (2010) Greenhouse warming and the 21^st^ century hydroclimate of southwestern North America. Proceedings of the National Academy of Sciences 107: 21277–21282. doi: 10.1073/pnas.0910856107 10.1073/pnas.0910856107 PMC3003097 21149692

[B75] SEMARNAT (Secretaría de Medio Ambiente y Recursos Naturales) (2010) Norma Oficial Mexicana NOM-059-Ecol-2010. Protección ambiental-Especies nativas de México de flora y fauna silvestres-Categorías de riesgo y especificaciones para su inclusión, exclusión o cambio-Lista de especies en riesgo. Diario oficial (Segunda Sección, 30-dic), 77 pp http://www.profepa.gob.mx/innovaportal/file/435/1/NOM_059_SEMARNAT_2010.pdf

[B76] Servoss JM Sferra S Jones TR Sredl MJ Rosen PC (2013) Geographic Distribution: *Ramphotyphlops braminus*. Herpetological Review 44: 477.

[B77] Smith GR Lemos-Espinal JA (2015) Herpetofaunal density of the United States-Mexico Border States, with comparison among the states. In: Lemos-Espinal JA (Ed.) Amphibians and Reptiles of the US-Mexico Border States. Texas A&M University Press, College Station, 196–205.

[B78] Smith HM (1934) Descriptions of New Lizards of the Genus *Sceloporus* from Mexico and Southern United States. Transactions of the Kansas Academy of Science 37: 263–285. doi: 10.2307/3625310

[B79] Smith HM (1942) Summary of the collections of snakes and crocodilians made in Mexico under the Walter Rathbone Bacon Traveling Scholarship. Proceedings of the United States National Museum 93: 393–504. doi: 10.5479/si.00963801.93-3169.393

[B80] Smith HM (1944) Snakes of the Hoogstraal Expeditions to northern Mexico. Zoological Series, Field Museum of Natural History 29: 135–152.

[B81] Smith HM (1951) A new species of *Leiolopisma* (Reptilia: Sauria) from Mexico. University of Kansas Science Bulletin 34(3): 195–200.

[B82] Smith HM Hall WP (1974) Contributions to the concepts of reproductive cycles and the systematics of the *scalaris* group of the lizard genus *Sceloporus*. Great Basin Naturalist 34: 97–104. doi: 10.5962/bhl.part.15512

[B83] Taylor EH (1939) New species of Mexican anuran. University of Kansas Science Bulletin 26: 385–405.

[B84] Taylor EH (1940) Two new anuran amphibians from Mexico. Proceedings of the United States National Museum 89: 43–47. doi: 10.5479/si.00963801.89-3093.43

[B85] Taylor EH (1941) Two new species of Mexican plethodontid salamanders. Proceedings of the Biological Society of Washington 54: 81–85. http://www.biodiversitylibrary.org/page/3459923#page/105/mode/1up

[B86] Taylor EH Smith HM (1945) Summary of the collections of amphibians made in Mexico under the Walter Rathbone Bacon Traveling Scholarship. Proceedings of the United States National Museum 95: 521–613. doi: 10.5479/si.00963801.95-3185.521

[B87] Valdez-Tamez V Foroughbakhch R Alanís-Flores G (2003) Distribución relictual del Bosque Mesófilo de Montaña en el noreste de México. Ciencias UANL 4: 360–365. http://eprints.uanl.mx/1518/1/distribucion_relictual.pdf

[B88] Velazco-Macías CG (2009) Flora del Estado de Nuevo León, México: Diversidad y Análisis Espacio-Temporal. PhD Thesis, San Nicolás de Los Garza, Universidad Autónoma de Nuevo León, Mexico.

[B89] Velazco-Macías CG Foroughbakhch Pournavab R Álvarado Vázquez MA Alanís Flores GJ (2008) La familia Nymphaeaceae en el estado de Nuevo León. Journal of the Botanical Research Institute of Texas 2: 593–603. http://www.jstor.org/stable/41971677

[B90] Wilson LD Johnson JD Mata-Silva V (2013a) A conservation reassessment of the amphibians of Mexico based on the EVS measure. Amphibian & Reptile Conservation 7: 97–127. http://amphibian-reptile-conservation.org/pdfs/Volume/Vol_7_no_1/Special_Mexico_Issue_ARC_7_1_97-127_e69_high_res.pdf

[B91] Wilson LD Mata-Silva V Johnson JD (2013b) A conservation reassessment of the reptiles of Mexico based on the EVS measure. Amphibian & Reptile Conservation 7: 1–47. http://amphibian-reptile-conservation.org/pdfs/Volume/Vol_7_no_1/Special_Mexico_Issue_ARC_7_1_1-47_e61_high_res.pdf

